# Optimizing the image of fluorescence cholangiography using ICG: a systematic review and ex vivo experiments

**DOI:** 10.1007/s00464-018-6233-x

**Published:** 2018-05-18

**Authors:** Jacqueline van den Bos, Fokko P. Wieringa, Nicole D. Bouvy, Laurents P. S. Stassen

**Affiliations:** 1 Department of Surgery, Maastricht University Medical Center, PO box 616, 6200 MD Maastricht, The Netherlands; 2 School of Nutrition and Translational Research in Metabolism, Maastricht University, Maastricht, The Netherlands; 3 Faculty of Health Medicine and Life Sciences, Maastricht University, Maastricht, The Netherlands; 4 imec the Netherlands, Eindhoven, The Netherlands

**Keywords:** Indocyanine green, Near-infrared fluorescence imaging, NIRF, Fluorescence intensity

## Abstract

**Background:**

Though often only briefly described in the literature, there are clearly factors that have an influence on the fluorescence intensity, and thereby the usefulness of the technique. This article aims to provide an overview of the factors influencing the fluorescence intensity of fluorescence imaging with Indocyanine green, primarily focussed on NIRF guided cholangiography.

**Methods:**

A systematic search was conducted to gain an overview of currently used methods in NIRF imaging in laparoscopic cholecystectomies. Relevant literature was searched to gain advice on what methods to use. Ex vivo experiments were performed to assess various factors that influence fluorescence intensity and whether the found clinical advices can be confirmed.

**Results:**

ICG is currently the most widely applied fluorescent dye. Optimal ICG concentration lies between 0.00195 and 0.025 mg/ml, and this dose should be given as early as achievable—but maximum 24 h—before surgery. When holding the laparoscope closer and perpendicular to the dye, the signal is most intense. In patients with a higher BMI and/or cholecystitis, fluorescence intensity is lower, but NIRF seems to be more helpful. There are differences between various marketed fluorescence systems. Also, no uniform method to assess fluorescence intensity is available yet.

**Conclusions:**

This study identified and discussed several factors that influence the signal of fluorescence cholangiography. These factors should be taken into account when using NIRF cholangiography. Also, surgeons should be aware of new dyes and clinical systems, in order to benefit most from the potential of NIRF imaging.

**Electronic supplementary material:**

The online version of this article (10.1007/s00464-018-6233-x) contains supplementary material, which is available to authorized users.

The most common laparoscopic procedure in the Netherlands is laparoscopic cholecystectomy (LC), which is performed 23,000 times each year [1]. The most feared complication in this surgery is bile duct injury. Even though LC is a very common procedure, bile duct injury still has an incidence of 0.3–0.7% [2–5]. Generally, bile duct injury leads to bile leakage, causing abdominal sepsis. It can also lead to obstruction, with obstructive jaundice, eventually potentially leading to a need for liver transplantation in the worst case [6]. Late recognition is common in bile duct injuries, resulting in significant morbidity and mortality, a lower quality of life and extra costs [6–9]. The main cause for bile duct injury is misidentification of the anatomy [10, 11]. Therefore, techniques to improve the visualization of the anatomy are desired.

Several imaging techniques to improve recognition of the relevant anatomical structures have been proposed. One is intra-operative cholangiography. Although this imaging technique is advised to reduce the risk of bile duct injury, there are disadvantages such as radiation exposure and need for additional equipment, knowledge and personnel to perform this procedure and extra costs [2, 11–13]. Because of these disadvantages, there is no worldwide consensus about implementation of intra-operative cholangiography [14].

Another technique to help identify the relevant anatomical structures is the use of near-infrared fluorescence (NIRF) imaging. This is a relatively new and promising technique to improve recognition of the biliary anatomy by injecting a fluorescent dye [15, 16]. The clinically used dye is Indocyanine green (ICG), belonging to the family of cyanine dyes [17]. ICG is a water-soluble tricarbocyanine with a molecular weight of 774.96 absorbing excitation light at 800–835 nm while emitting fluorescent light at 740–793 nm [18–20]. Generally, ICG is considered safe with a very low incidence of complications, although there is a possibility of an allergic reaction to the sodium-iodine in the ICG [21–23]. Other dyes are not generally available yet, either because they are not excreted in bile or because they are still in a research phase and thus not yet available for routine clinical use [24, 25].

In addition to this small risk for an allergic reaction, all researchers and surgeons working with near-infrared (NIR) fluorescence-imaging experience limitations of the technique. Though often only briefly described in the literature, there are clearly factors such as the given dose and distance between the laparoscope and the target that have an influence on the fluorescence intensity, and thereby the usefulness of the technique. Since this technique is nowadays commonly used, it is of importance to obtain more general knowledge about the factors that influence the fluorescence intensity. Therefore, this article aims to provide an overview of these factors influencing the fluorescence intensity, primarily focussed on NIRF guided cholangiography during laparoscopic cholecystectomy.

## Materials and methods

To provide an overview of the factors influencing the fluorescence intensity in NIRF-guided cholangiography during laparoscopic cholecystectomy, three strategies were combined. First a systematic review of the literature was performed. Secondly, using free searches, more information about specific suspected factors of influence was sought. Thirdly, factors of influence identified from the literature were further investigated with ex vivo experiments.

Since no (new) patients are included in this study, IRB approval or patients informed consent was needed for this study.

### Literature search

A Pubmed search on methods used for fluorescence cholangiography and its influencing factors was performed by two reviewers through November 2017. Used search terms were as follows: ((((((((((((near-infrared imaging) OR fluorescence imaging) OR fluorescent imaging) OR near-infrared fluorescence) OR fluorescent dyes) OR fluorescent dye) OR “Fluorescent Dyes“[Mesh]) OR ICG) OR infracyanine green) OR indocyanine green) OR “Indocyanine Green“[Mesh])) AND ((((((“Cholecystectomy“[Mesh]) OR Cholecystectomy) OR laparoscopic cholecystectomy) OR “Cholecystectomy, Laparoscopic“[Mesh]) OR gallbladder removal) OR laparoscopic cholecystectomies). No Language restrictions were applied. Studies presenting original data on performing near-infrared fluorescence-guided cholecystectomy in human subjects were included. Therefore animal studies, protocol-articles, reviews and case reports were excluded. From the included reports, data regarding the used methods and outcomes (visibility of anatomical structures) was extracted.

### Ex vivo experiments

In addition, ex vivo experiments were performed to further analyse certain aspects. The aim of these experiments was to evaluate whether albumin is needed in the medium used for ICG to become fluorescent and to study the influence of the concentration of ICG, the distance between the laparoscope and the target, the angle between the laparoscope and the target and the penetration depth on the fluorescent signal.

A laparoscopic Near-infrared Fluorescence Imaging System (Karl Storz GmbH, Germany) was used, as we use in daily clinical practice. This system includes a D-light P xenon light source, an IMAGE 1 camera and a 30 degree 10 mm fluorescence laparoscope (Karl Storz GmbH, Germany).

Experiments were performed both with ICG diluted in NaCl 0.9% and with ICG dilution in 35 mg/ml albumin in a 0.9% NaCl dilution. This was done as ICG is considered in vivo to bind to albumin. The aim was to assess whether such binding ex vivo was necessary in order to obtain a signal.

Initially, we used ten different concentrations of ICG, ranging between 10 and 0.125 mg/ml. In these experiments, a lower concentration gave a better signal. To find the optimum, eventually twenty-one solutions ranging from 10 to 0.000121875 mg/ml were used. From each dilution, 9 times 1 ml of the ICG was placed on a wells plate.

The influence of distance was measured holding the endoscope at distances from 14 to 1 cm from the surface of the dye. Also, the maximum distance at which the fluorescence could still be detected was measured for each concentration.

The influence of angle between the laparoscope and the surface of the dye was measured by comparing the middle cup, at which the laparoscope was aimed, with the surrounding cups with ICG and by holding the endoscope at different angles.

**Fig. 1:**
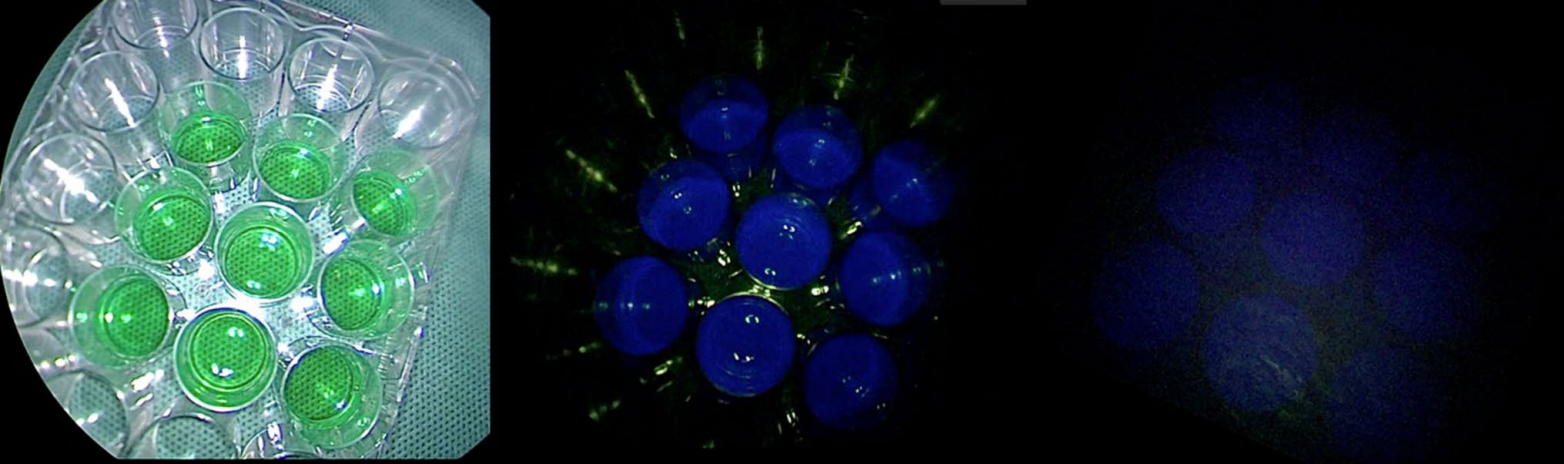
Setup ex vivo Experiments. *Legend* 9 times 1 ml of ICG in a dilution of 0.125 mg/ml in the cups of a Wells plate. Left: in white light. Middle: in NIRF light. Right: in NIRF light with one plate (0.9 mm) of beeswax on top of the Wells plate

The penetration depth was evaluated with the use of beeswax plates stacked to different thicknesses. This medium was chosen because it approaches the scattering behaviour and translucent light penetration of human tissue quite well [26]. In fact, from the renaissance until the early twentieth century, anatomical wax models were used for medical education and until today provide an impressive visual display [27]. Beeswax plates of exactly 0.9 mm thickness were one by one progressively stacked on the wells plate to stepwise increase the thickness of scattering material on top of the fluorescent dye. In all experiments, fluorescence intensity was measured using Osirix (version Lite 8.5.2, Pixmeo). The setup of the ex vivo experiment is shown in Fig. 1.

Additional information for this article was retrieved by a free search in the PubMed database, hand-searches of the retrieved references and personal experience with the technique.

## Results

**Fig. 2:**
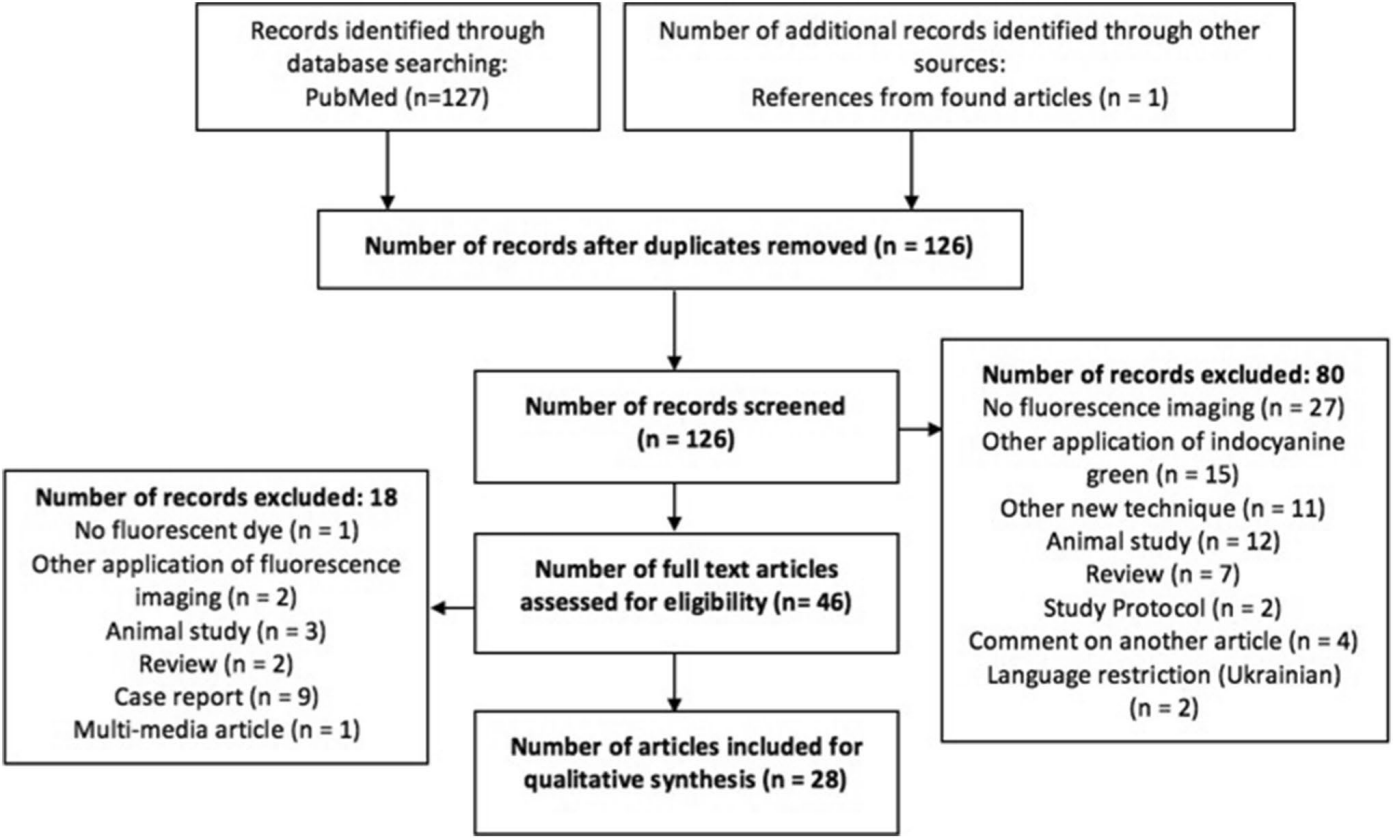
Flowchart of systematic search

The systematic search was performed to retrieve insight in the currently used methods of performing near-infrared fluorescence guided cholangiography and the factors influencing its signal intensity. This search resulted initially in 126 articles, of which 80 were excluded based on title and abstract and 18 more after full text assessment. 28 articles were included in this review. Figure 2 presents the flowchart of this search [13, 19, 28–53]. An overview of the included articles and their results is given in Tables 1 and 2 (Supplementary Material), respectively. The results of literature search and the results of the performed experiments will be discussed in this section.

### Fluorescent dye and concentration

In all studies, ICG is used as the fluorescent dye. Both *Indo*cyanine green and *Infra*cyanine green where used. ICG was administered intravenously, except in the study by Graves et al. [29] and Liu et al. [53], who injected the fluorescent dye directly into the gallbladder. Graves et al. made a special solution for this intra-gallbladder injection. First, a 0.25 mg/ml solution of ICG in sterile water was created. Once the gallbladder fundus was grasped and retracted, a needle-tipped Kumar cholangiogram catheter (Nashville Surgical Instruments, Springfield, TN) was introduced through the 12-mm port and used to puncture the infundibulum of the gallbladder. 9 ml of bile was aspirated from the gallbladder through this catheter and mixed with the 0.25 mg/ml ICG solution. The new 0.025 mg/ml ICG-bile solution was reinjected into the gallbladder. The authors describe an immediately glow-up of the solution with no lag time, quickly filling the gallbladder and through the cystic duct the extrahepatic ducts. Due to the absence of background fluorescence from the liver, improved visual contrast was claimed as compared to intravenous injection [29]. Liu et al. used a less complicated method, in which the ICG was diluted to a concentration of 0.125 mg/mL. 10 mL of this ICG dilution was injected through a gallbladder drain when this was already in situ, or through a Veress needle after draining the gallbladder from bile [53]. In these patients, although background fluorescence was absent, results comparable to intravenous administration were found.

The most commonly used doses of ICG for intravenous administration where a fixed dose of 2.5 mg bolus (in 13 of the studies) or a dose adjusted to the patient’s weight of 0.05 mg/kg (in 6 of the included studies). Other used doses are fixed dosed of 0.25, 3.75, 5, 10 and 12.5 mg and weight-adjusted doses of 0.1–0.4, 0.2, 0.4 and 0.5 mg/kg (see Table 1 in Supplementary Material). When using a 2.5 mg bolus, the cystic duct was visualized in an average of 94% patients. When using 0.05 mg/kg, the average percentage of visualization of the cystic duct was 98%.

Zarrinpar et al. [54] undertook a study with systematic variation of dosing (and timing) of injection of ICG. In 37 patients, the given dose varied between 0.02 and 0.25 mg/kg. With an increasing dose, the visualization of the extrahepatic biliary tract improved. No decrease at the highest doses was observed. Boogerd et al. [30] compared a dose of 5 and 10 mg with optimal signal at the lower dose of 5 mg.

**Fig. 3:**
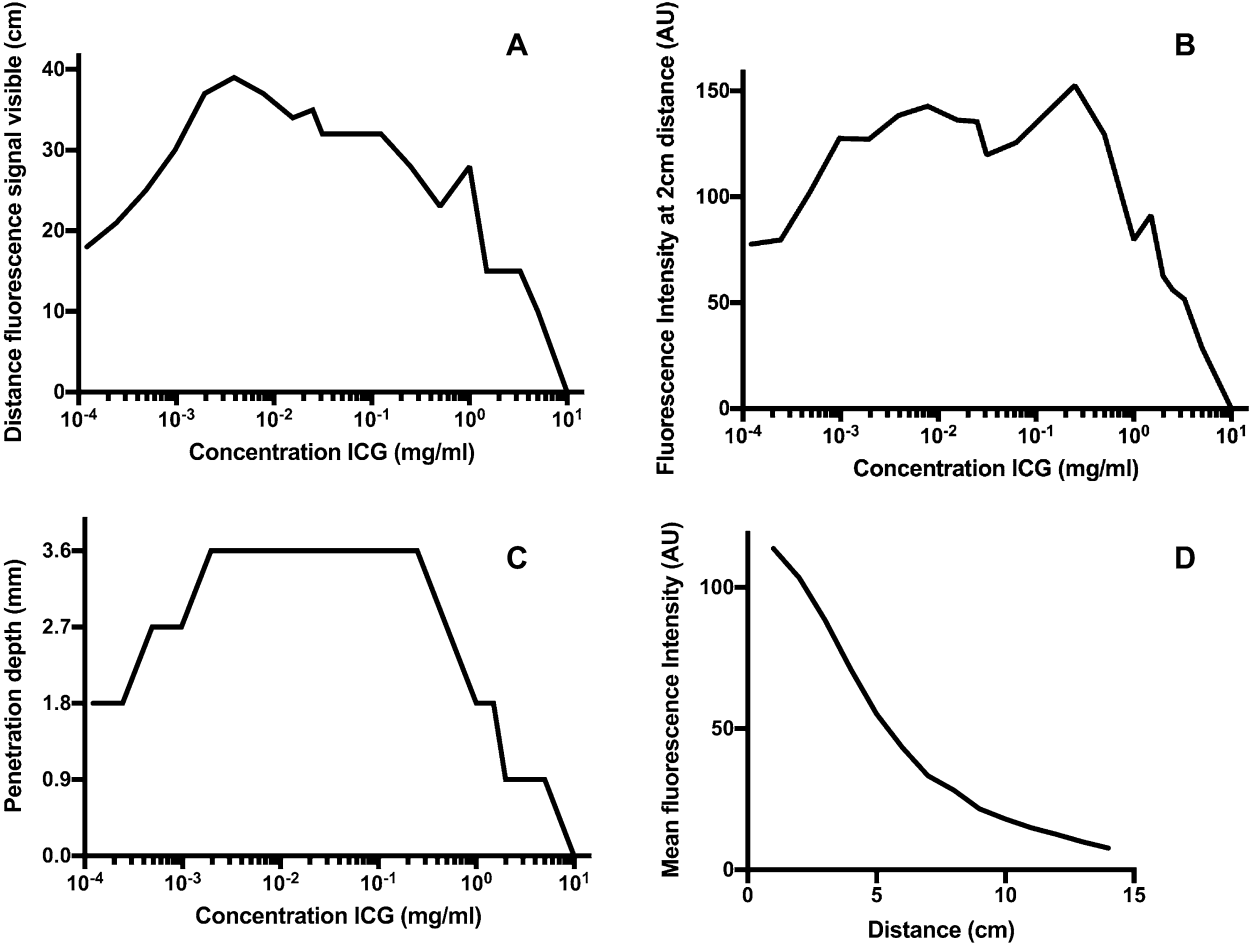
Results ex vivo experiments. *Legend* **A** Maximum distance of visibility fluorescent signal for the tested concentrations. **B** Fluorescence intensity at 2 cm distance for the tested concentrations. **C** Maximum penetration depth of fluorescent signal for the tested concentrations. **D** Fluorescence intensity at increased distance between laparoscope and dye-surface

The results of our ex vivo experiments are depicted in Fig. 3. When ICG was diluted in NaCl 0.9%, no fluorescent signal could be obtained. Therefore, all further experiments were performed with ICG diluted in 35 mg/ml albumin in a 0.9%NaCl solution. The optimum concentration in which the highest measured fluorescence intensity at 2 cm distance was between 0.00195 and 0.025 mg/ml. A concentration of 0.0039 mg/ml would be a dose of 19.5 mg to obtain this ideal concentration in the blood in an average patient with 5l blood, which is much higher than current used concentrations. However, when using this technique in laparoscopic cholecystectomy, we would want the concentration of 0.0039 mg/ml in the cystic duct. With a content of 60 ml of bile in the gallbladder, a dose of 0.234 mg would then be desirable assuming all ICG is cleared by the liver.

The used concentration of the administered ICG solution was not clearly described in every study. The studies that do provide information on ICG concentration use 2.5 mg/ml [19, 33, 34, 40, 46, 49–52]. In all, except two studies, the fluorescent dye was administered intravenously.

### Timing of administration

The timing of administration differed among the studies and ranged mostly between 1 and 2 h and 15 min before surgery. Verbeek et al. [55] investigated whether a longer period between the administration of ICG and the imaging resulted in an improved visibility of the biliary anatomy. They found that application 24 h prior to surgery does result in a significant better signal-to-background ratio [55]. This is mainly caused by a lesser fluorescent signal coming from the liver. Kono et al. [56] found a significant difference in interval between injection and imaging between patients in whom the cystic duct-common hepatic duct confluence could be visualized by NIRF imaging and the patients in whom this could not, with median intervals of 90 (15–65) minutes and 47 (21–205) minutes, respectively. The above is in line with the advice of Boogerd et al. [30], for an optimal time interval of 3 h between administration of ICG and intra-operative fluorescence cholangiography.

### Penetration depth

Circumstances that result in thicker tissue covering the structures to identify are obesity and cholecystitis. These require a higher penetration depth of the signal.

Average BMI in the included studies ranged between 20.4 and 32.3 kg/m^2^ with a weighted average of 30.0 kg/m^2^ for all studies who report mean BMI. Dip et al. [35] specifically investigated the accuracy of NIRF-guided surgery in morbidly obese subjects undergoing laparoscopic cholecystectomy. No differences in hepatic duct, common bile duct and accessory duct visualization were detected in the obese and non-obese groups (*p* value 0.09, 0.16 and 0.66, respectively). Imagi et al. [34] state that obesity is an important factor that can prevent identification of biliary structures under fluorescent cholangiography. Other studies notice that when not all aimed structures where visualized under fluorescent cholangiography in every patient, the patients in which the structures were not visible were all obese [40, 42, 44, 51]. Ankersmit et al. [31], however, found no differences in ability to visualize the biliary structures in obese patients. Their hypothesis is that other patient and surgical-related factors, including inflamed tissue, may interfere with and influence the success rate of fluorescence visualization of the bile ducts in complicated cases [31].

Of the included patients in the studies found, 17% had acute cholecystitis. Four of the studies make a comment about the influence of cholecystitis on the usefulness of the NIRF technique. Zroback et al. [33] note that in their experience, during dissection of the most inflamed gallbladders was when the technology was most appreciated, but no specific remark is made on the hindrance by thickened tissue. The other articles mention that the patients in which the biliary structures were not visible with NIRF more often had cholecystitis than the patients in which they were visible [13, 39, 42]. Although in several studies the indication for surgery was also cholecystitis and the number of patients with this condition was given, no specific comparison was made with regard to the successful imaging of the desired structures between the patients with and without cholecystitis in most studies. Liu et al. [53] make this comparison and conclude that in these patients, NIRF helped to significantly improve the visualization rates of the cystic duct in patients with cholecystitis compared to white light, whilst in cases with lithiasis without inflammation, the visualization rate of the cystic duct was similar in NIRF and white light.

In our ex vivo experiments, the penetration depth was studied at all concentrations separately. We observed a correlation between the thickness of material to be penetrated and the resulting signal, with an optimum and a maximum. A maximum penetration depth of 3.6 mm was measured. The optimum is at the same concentrations as we observed in our experiments on the concentration of ICG namely between 0.00195 and 0.025 mg/ml. See also Fig. 3.

### Distance to the target

Kono et al. [56] described a decrease by 50% of the contrast signal when the distance from the bile duct and tip of the laparoscope increased from 5 to 15 cm. However, other manuscripts do not clearly describe the distance used between the bile ducts and the laparoscope.

In our ex vivo experiments, a lower fluorescence intensity was found in all concentrations when the laparoscope was held at a greater distance. A 5 up to 30 times lower fluorescence intensity was observed when increasing the distance from 5 to 14 cm of the ICG solution, as is illustrated in Fig. 3. When looking at the different concentrations, the maximum distance at which the fluorescent signal was still detectable was higher at the ‘optimal concentrations’ as shown in Fig. 3.

### Angle between laparoscope and tissue

Kono et al. [56] point out that the tip of the laparoscope should be placed vertically to Calot’s triangle to directly irradiate exciting light on the bile ducts and obtain the optimal fluorescence signal. However, as with the distance of the laparoscope, other manuscripts do not mention whether the laparoscope was placed vertically or in another angle. Some of the articles use a zero-degree laparoscope, while others use a 30-degree laparoscope, which may be of influence on the angle of examination. In our experiments, a higher fluorescence intensity was found when holding the laparoscope more vertically towards the tissue.

### Differences between systems

The systems used in the included studies were 10 times a system by Karl Storz; 6 times the Firefly system; 3 times an Olympus system; 5 times a Hamamatsu system and 2 Stryker systems. Two studies did not mention which system was used. When comparing the studies using the different systems studies using Karl Storz can visualize the cystic duct in 93%; Firefly in 97% Olympus in 92% Hamanatsu in 92% and Stryker in 94%.

Kono et al. [56] compared five imaging systems. Namely, a prototype fluorescence imaging system from Hamamatsu Photonics (Hamamatsu, Japan and Shinko Optical, Tokyo, Japan), the system from Olympus Medical Systems (Tokyo, Japan), thirdly a high-definition model From Hamamatsu Photonics (Hamamatsu, Japan and Shinko Optical, Tokyo, Japan), the high-definition fluorescence imaging system from Karl Storz (Tuttlingen, Germany) and fifth, the high-definition imaging system from Novadaq (Toronto, Canada). A significant difference between these systems tested for signal contrast was found [56]. It is not clear whether the systems tested are exact alike with the systems that are at present commercially available and clinically used.

### Interpretation of the signal

Most studies only state whether a certain anatomical structure is visible or not, without quantification of the fluorescence intensity. There is no generally applied analytic measure to objectify the fluorescence intensity yet. When the fluorescence intensity is measured in literature, several methods based on the colour-intensity are used. In most of these methods, only the fluorescence intensity of the target is measured.

Schols et al. [43, 46] use in both articles measurement of the Target to Background Ratio (TBR) to objectify the degree of fluorescence illumination. For this, OsiriX 5.5.1 Imaging Software was used. The TBR was defined as the mean fluorescence intensity (FI) of two point regions of interest (ROIs) in the target (i.e. CBD, CD, or CA) minus the mean FI of two background ROIs in the liver hilum, divided by the mean FI of the two Background ROIs in the liver hilum; The used formula was as follows: TBR = (FI of target−FI of background)/FI of background.

Ashitate et al. [57] apply the same formula, but do not mention which software was used. Also, the exposed rectus muscle was used as background [57]. Objectively this is correct, as any consistent background can be chosen, but subjectively, it is more logical to use the liver as background, as this is the actual observed background during the operation.

Kono et al. [56] also use a signal to background ratio. In their series, Photoshop CS5 software (Adobe Systems, San Jose, CA) was used to calculate the fluorescence intensity in the regions of interest with a range of 0–255 from still images. The used formula here was as follows: Signal contrast = (FI in fluorescence regions−FI in background)/225.

Zarrinpar et al. [54] used ImageJ (US National Institutes of Health, Bethesda, MD; http://imagej.nih.gov/ij/) software to calculate intensity ratios. They divided the fluorescence intensity signal of the common bile duct by that of the surrounding fat or liver. Verbeek et al. [55] use the same formula as Zarrinpar, but do not mention which software was used.

## Discussion

The aim of this article is to give an overview of factors influencing the signal intensity during fluorescence imaging, primarily focussed on NIRF-guided cholangiography with ICG during laparoscopic cholecystectomy. These influences can be divided in patient factors, the applied fluorescent dye, the equipment and the method of assessing fluorescence intensity.

Regarding patient factors, obesity and inflammation, i.e. cholecystitis, are of importance. These may cause thickening of tissue covering the structures to be visualized, thereby decreasing signal intensity. Although fluorescence imaging has a better tissue penetration than white light, due to its emission in the near-infrared wavelength zone, penetration is limited by such factors.

In several studies, patients with cholecystitis were included. In four studies, a comment is given about the influence of cholecystitis on the usefulness of the NIRF technique. Zroback et al. [33] appreciated the NIRF technology most while dissecting the most inflamed gallbladders. On the contrary, three other authors mention that in patients in whom the biliary structures could not be visualized, more often cholecystitis was present than in patients in whom imaging was successful [13, 39, 42]. Liu et al. report a significant difference in visibility of the cystic duct with NIRF compared to white light in patients with cholecystitis [53]. This is an indication that NIRF imaging might be more helpful in cholecystitis patients. No difference in complications due to NIRF imaging was reported between patients with and without cholecystitis.

Obesity may result in more fatty tissue overlying the extrahepatic biliary tree. The average BMI in the included studies ranged between 20.4 and 32.3 kg/m^2^ with an overall average of 30.0 kg/m^2^. Considering this high average BMI and the good results the manuscripts describe; a high BMI seems not to be a burden in the use of NIRF cholangiography. Dip et al. [35] specifically investigated the accuracy of NIRF guided surgery in morbidly obese subjects undergoing laparoscopic cholecystectomy and could not find differences in biliary duct visualization between the obese and the non-obese groups [35]. Some studies confirm this absence of influence of BMI on the visualization scores [54, 56]. On the contrary, other studies claim that BMI has a negative influence on the visibility of the structures using NIRF [34, 40, 42, 44, 51]. It is even suggested that also a more common inflammatory response is present in these patients [42]. The practice of using fluorescence cholangiography is that it is applied while gradually dissecting the surgical area. Sooner or later during this dissection, NIRF will lead to confirmation of the anatomical structures. More fatty tissue requires more dissection and—most likely—later visualization of structures. The earlier or later recognition may influence the surgeon’s satisfaction with the NIRF technique. Another explanation for differences between studies may be that the signal of ICG depends on the concentration used [58]. It is possible that in patients with a higher bodyweight, the concentration of ICG differs when using a fixed and not a weight-adjusted dose. This will be discussed hereunder.

In the performed ex vivo experiments, a maximum penetration depth of 3.6 mm was achieved. This confirms the limits of penetration of the NIRF signal. However, bees wax was used to measure the penetration depth. Although spectral properties are similar to human tissue, it is possible that the penetration depth in human tissue is different. Anyhow, this in vitro method at least offers an objective and simple tool to compare the performance of different laparoscopes, light sources, and camera systems.

In conclusion, NIRF seems also to be beneficial in circumstances where penetration depth is limited due to thickened overlying tissue. In these cases, the image will be obtained later, after relatively more dissection has been performed. The surgeon should be aware of this, but with this in mind, can use the technique to enhance recognition of the essential structures.

Concerning the fluorescent dye used, several factors may be of influence on the signal obtained: the type of dye, the dose used and its concentration, the timing and the route of administration.

Regarding the type of dye, there are several factors that determine the effectiveness of a fluorescent dye. These are probe targeting (a dye for bile duct imaging should be able to reach the bile ducts and to some extent accumulate there), activation, pharma kinetics (how fast is the dye at the desired place and how long will it stay there?), biocompatibility and photo physics [59]. The dye should ideally be in the near-infrared range, since this offers the spectral range with best light penetration (so-called near-infrared window). Also the use of a near-infrared filter and light source eliminate auto-fluorescence [60]. Wavelengths below 600 nm encounter this higher auto-fluorescence due to the presence of many endogenous fluorophores and strong scattering. Below 600 nm, there is a strong absorbance of haemoproteins such as haemoglobin, myoglobin and cytochromes causing less deep tissue penetration [59, 61].

Indocyanine green is currently the only clinically used dye for fluorescence cholangiography, illustrated by the fact that it was used in all 27 studies of this review. In most of these studies, ICG was mostly an abbreviation for *indo*cyanine green, but in four studies the iodine-free *infra*cyanine green was used. The absence of iodine in infracyanine seems to make it less toxic and available for patients with an iodine allergy. We did not perform in vitro experiments with infracyanine green, but the absorption and emission spectra of both dyes do not differ [62], and thus the signal is expected to be equivalent.

Another previously tested fluorescent dye is Methylene Blue (MB). This has not been used in the studies reported. MB and ICG have been compared in an animal study using pigs in which both dyes were directly injected into the gallbladder through a 10 Fr catheter that was inserted into the gallbladder fundus [63]. Using this method, ICG had a higher target to background signal, due to a higher extinction coefficient and higher fluorescence rate. An advantage of MB is limited liver uptake resulting in less background signal. The signal becomes visible within minutes and remains adequate for 120 min [63]. A disadvantage of MB is the absorption and emission around 700 nm which is subject to a higher background auto-fluorescence [63] but also requires different settings of the equipment. Another drawback is the need for intra-gallbladder administration of the dye, since methylene blue is cleared by the kidneys, not the liver [64]. Ashitate et al. [57] combined two dyes in an animal study. Herein, they compared three different combinations; first methylene blue for visualization of the cystic artery and indocyanine green for visualization of the bile duct; secondly ICG for the artery and Methylene blue for the bile duct; thirdly ZW-800-1 for the artery and methylene blue for the bile duct. The authors’ conclusion is that the third combination had the best overall performance, and the second combination was the best combination of clinically available dyes [57].

Apart from the above mentioned ZW-800, other new preclinical dyes such as VM678, IRDye®800CW, IRDye®800BK and IRDye®800NOS have been tested in animals and show very promising results [24, 65]. These are due to better pharmacokinetic characteristics and target-to-background ratio of these dyes [24, 66]. The IRDyes have the same spectral characteristics as ICG, which enables the use of the same equipment. IRDye®800CW is expensive, with a price tenfold that of ICG; IRDye®800BK and IRDye®800NOS however cost the same as ICG [65]. But these dyes are not yet FDA approved.

In conclusion, ICG is currently the most used and preferred dye. Only limited data are available on MB and it is not used in clinical practice for cholangiography, probably based on the characteristics described above. New preclinical dyes are promising but await approval for clinical practice.

The dose used may also be of influence on the performance of the dye. In the studies presented, a bolus was given (2.5 mg in 13 studies, 12.5 mg and 3.75 mg in two others); or a weight-adjusted dose (0.05 mg/kg in seven studies and 0.04 mg/kg in three other studies).

Zarrinpar et al. [54] undertook a prospective study with systematic variation of dosing and timing from injection of ICG to visualization. In 37 patients, doses were given varying between 0.02 and 0.25 mg/kg. Their conclusion was that with increasing dose, the visualization of the extrahepatic biliary tract improved. Visualization was also better with increased time after ICG administration (10 min vs. 45 min vs. 3 h). Boogerd et al. [30] compared a dose of 5 and 10 mg and advise to use the lower dose of 5 mg.

Our ex vivo experiments suggest that the 2.5 mg bolus that is used in most studies, is probably below the optimal clinical dose. One should however take into account that these ex vivo experiments are a controlled setting, which is not entirely comparable to the complex in vivo biological system in which we use the NIRF cholangiography. Therefore, a recommendation regarding the optimal dose cannot be given based on these experiments. Nevertheless, the conclusion can be drawn that applying a weight-adjusted dose seems preferable over a fixed dose.

The used concentration of the administered ICG solution was not clearly described in every study. The studies that describe the used concentration all use 2.5 mg/ml [19, 33, 34, 40, 46, 49–52]. This is remarkable since the leaflet that comes with ICG advises to dilute the ICG to a solution of 5 mg/ml. However, a concentration of 2.5 mg/ml is more feasible in daily practice since 1 ml can be retrieved more precise than just 0.5 ml.

Hiratoglou et al. [62] investigated the influence of the concentration and solvent medium on the light-absorbing prosperities in ICG. When glucose 5% was used as a solvent medium, the absorption between 600 and 700 nm was decreased, compared with the absorption with Balanced Salt Solution (BSS) or BSS Plus. These differences between BSS/BSS Plus- and glucose 5%-diluted ICG decreased when the concentration was lowered. They conclude that depending on the used solvent medium, the absorption spectrum of ICG changes with different concentrations [62]. Based on these findings, it might be advisable to use sterile water as a solvent medium, as most groups currently do, and not a glucose solution.

Kono et al. [56] found a lower fluorescence intensity when using a more diluted solution of ICG.

Our ex vivo experiments show an optimum of ICG concentration. This optimum seems to be at a higher concentration than is currently achieved in the clinical situation. However, this conclusion is an extrapolation of the experimental data to the clinical situation for the concentration of ICG in the blood. The extrapolation may not be correct and is also not directly translatable to the concentration in the bile ducts. And one must be aware that not so much the concentration of the solution applied, but the total dose administered determines the systemic concentration of the dye.

The timing of administration ranged mostly between 2 h and 15 min before surgery. Verbeek et al. [55] investigated whether a longer time between the administration of ICG resulted in an improved visibility of the biliary anatomy. The authors found that administration 24 h prior to surgery results in a significantly better signal to background ratio [55]. This is mainly caused by a lesser fluorescent signal coming from the liver. A problem with this approach is the practical applicability: in day care admissions and in acute cholecystitis an injection 24 h before surgery often is not feasible. Also, since only 30 min versus 24 h before surgery were tested, the optimum may lie between these timeslots. Kono et al. [56] and Boogerd et al. [30] also conclude that earlier administration results in better visualization. In conclusion, administration closer to the time of surgery can result in a more prominent background signal from the liver. Therefore, administration as early as possible (but not earlier than 24 h) should be considered.

In all studies, except for the study by Graves et al. [30] and Liu et al. [53], the fluorescent dye was administered intravenously. This is easy and convenient for the patient, since intravenous access is needed anyway for induction of anaesthesia. However, systemic injection results in the disadvantage of occurrence of (background) fluorescence of the liver, which considerably decreases the signal to background ratio. Another limitation of this administration route is the dependency on the refilling of the gallbladder and cystic duct with fluorescent bile by reflux, which is unpredictable [38]. A solution might be direct injection of ICG into the gallbladder. Lui et al. [67] previously examined the feasibility of this technique in pigs. They conclude that fluorescence cholangiography through direct intra-gallbladder injection can rapidly provide an adequate visualization of the gallbladder neck and cystic duct. The problems with a high background signal from the liver and the needed time for the ICG to be extracted from the blood and secreted into the biliary system are solved by this administration route. This is in line with their newer study in humans [53] and the study by Graves et al. [10], who injected the Indocyanine green in eight patients and achieved comparable encouraging results as described in the animal study. However, spill of ICG at the puncture site of the gallbladder may decrease overall visibility of the structures to be visualized. This is illustrated by the remark of Osayi [40] that in three of the patients the visualization was impaired due to bile leakage from the gallbladder, albeit that this was not caused by puncture, but by surgical trauma.

With regard to the imaging system, the distance and the angle between the endoscope and the target, and the properties of the system, have influence on the signal.

The distance between the fluorescent dye and the laparoscope seems to be of influence on the fluorescence intensity. Kono et al. [56] describe a decrease by 50% of the signal when the distance between the laparoscope and the target increased from 5 to 15 cm. Other manuscripts do not describe the distance between the bile ducts and the laparoscope. In our experiments, a clear relation was found between the distance of the laparoscope to the target and the measured fluorescence intensity (Fig. 3). In practice, the distance at which the endoscope is held, is primarily dictated by the optimal overview for surgical dissection. Ideally, the properties of the dye and the system enable the surgeon to maintain the same distance for dissection and for NIRF imaging. However, the surgeon must be aware that for optimal fluorescence signal temporarily adjusting of the distance may be necessary.

Kono et al. [56] point out that the tip of the laparoscope should be held vertically to Calot’s triangle to directly irradiate exciting light on the bile ducts to efficiently obtain fluorescence signals. However, as with the distance of the laparoscope, other manuscripts do not mention whether the laparoscope was held vertically or in another angle. Some of the articles use a zero-degree laparoscope, while others use a 30-degree laparoscope, which is also a factor that should be kept in mind when aiming for the optimal angle.

In the ex vivo experiments, a better signal was seen when the laparoscope’s central viewing axis was aligned perpendicular to the target (i.e. an angle between the tip of the laparoscope and the fluid level in het Well’s plate of 90 degrees). Because a 30-degree laparoscope was used, this needed to be held at 120 degrees to obtain the highest fluorescence intensity. This confirms the clinical findings of Kono et al. [56]. As with the distance, the position of the endoscope is mainly aimed at facilitating dissection. At moments of attention to the NIRF signal, focus should also be on the 90-degree angle to the target.

Five different systems were used in the included studies. As discussed before, the tissue depth to which NIRF imaging can detect agents is dependent upon their brightness and the sensitivity of the device [68]. The main components of a fluorescent imaging device are a spectrally adjusted light source for exciting ICG; optical filters for separation of the emitted fluorescent light and the background scattering and ambient light; camera(s) for detecting the emitted fluorescent signals; display software and display hardware [69]. The performance of a device is mainly determined by these components [68].

The most used light source type in the included studies is a xenon light for laparoscopy, and a laser diode in the robotic cases. Laser diodes enable the greatest sensitivity since the background due to background excitation light is the lowest. A xenon light source has a very broad bandwidth and has therefore a lower sensitivity [68]. The detection sensitivity in all fluorescent imaging systems is limited by the background signals. There is a spectral overlap between the backscattered excitation light and emitted fluorescence signals. In this way, non-fluorescent signals can be seen as fluorescence. Another problem is a limited optical density that allows passage of a small amount of ambient light, also called filter light leakage. This causes a ‘noise floor’, which makes it less probable that a device can detect small amounts of the fluorescent dye in tissues [68, 70]. A sufficient level of noise floor is needed to identify tissues of the surrounding, but when the noise floor is close to the fluorescent signal, the sensitivity of the imaging device and depth penetration of the signal will be reduced. A way to solve the issue of balancing between visualizing the surroundings and signal sensitivity is to superimpose images in real time by using separate cameras for reflectance and fluorescence [69, 71].

Kono et al. [56] performed the only comparative study to date with five laparoscopy systems for NIRF during cholecystectomy: Hamamatsu Photonics, Hamamatsu, Japan and Shinko Optical, Tokyo, Japan; Olympus Medical Systems, Tokyo, Japan; a high-definition unnamed model Japanese system; Karl Storz, Tuttlingen, Germany; Novadaq, Toronto, Canada. According to this study, the signal contrast was significantly different among the laparoscopic systems used for fluorescence imaging. In the meantime, since this study, systems and their properties have changed. The surgeon should be aware that differences exist which may influence the choice for a system.

Finally, it is important to evaluate the way the fluorescence intensity is assessed. Most studies only state whether a certain anatomical structure is visible or not, without quantitative measurement of the fluorescence intensity. There is no uniformly agreed analytic measure to objectify the fluorescence intensity. In most methods, only the fluorescence intensity of the target is measured, and not the fluorescence of the background.

The target-to-background ratio (TBR) is determined used by some authors Schols et al. [43, 46], Ashitate et al. [57], Kono et al [56], Zarrinpar [45], Verbeek [48]. Unfortunately, formulas that are used to calculate the ratio, the backgrounds chosen and the software used for measurement of the signal are not uniformly. In practice, the absolute signal and the subjective observation by the surgeon is what determines the clinical usefulness of the NIRF image. But for research purposes and comparison between studies an objective, uniform and validated quantification is necessary, which, as illustrated by the differences described, is not available yet. Our in vitro setup offers a simple and very cheap method for objective comparison between different system, but it would be recommendable that this topic is further refined and preferably standardized (e.g. by IEC).

The present review has some limitations. The majority of the studies are non-comparative. Especially to fulfil the purpose of the review, i.e. identify factors that influence the fluorescent image, such comparison would have been preferable. To partially address this drawback, ex vivo experiments were added to the review. In vivo studies would have been preferable, including the effect of human or animal tissue and circumstances on the acting of the fluorescent dye.

In conclusion, this study identified and discussed several factors that influence the signal of fluorescence cholangiography. Some are patient-specific and cannot be altered, such as BMI and cholecystitis. Even with a higher BMI and the presence of inflammation, NIRF is often successful. Other factors can be controlled by the surgeon. To maximize the fluorescence intensity during laparoscopic cholecystectomies, a weight-adjusted dose seems preferable over a fixed dose, especially in overweight patients; administration of this dose should take place as long as possible (max 24 h) before surgery; the laparoscope should be held at a right angle and close to the target tissue. Surgeons should be aware of new dyes that may come to the market and of differences in the properties of systems used, in order to benefit most of the potential of NIRF imaging. Further research should focus on the possibility of intra-gallbladder injection of ICG, other fluorescent dyes with a higher fluorescence intensity and faster clearance from the liver and the validation of a method to objectify the degree of fluorescence illumination in order to enable comparison between studies.

## Electronic supplementary material

Below is the link to the electronic supplementary material.

Supplementary material 1 (DOCX 49 KB)

Supplementary material 2 (DOCX 136 KB)

## References

[1] Statistiek CBvd (2010) Operaties in het ziekenhuis; soort opname, leeftijd en geslacht, 1995–2010 2010 [updated 05-02-2014; cited 2015. Available from: http://statline.cbs.nl/StatWeb/publication/?VW=T&DM=SLNL&PA=80386NED&LA=NL

[2] Flum DR, Dellinger EP, Cheadle A, Chan L, Koepsell T (2003). Intraoperative cholangiography and risk of common bile duct injury during cholecystectomy. JAMA.

[3] Fletcher DR, Hobbs MS, Tan P, Valinsky LJ, Hockey RL, Pikora TJ (1999). Complications of cholecystectomy: risks of the laparoscopic approach and protective effects of operative cholangiography: a population-based study. Ann Surg.

[4] Nuzzo G, Giuliante F, Giovannini I, Ardito F, D’Acapito F, Vellone M (2005). Bile duct injury during laparoscopic cholecystectomy: results of an Italian national survey on 56 591 cholecystectomies. Arch Surg.

[5] Waage A, Nilsson M (2006). Iatrogenic bile duct injury: a population-based study of 152 776 cholecystectomies in the Swedish Inpatient Registry. Arch Surg.

[6] Booij KA, de Reuver PR, Yap K, van Dieren S, van Delden OM, Rauws EA (2015). Morbidity and mortality after minor bile duct injury following laparoscopic cholecystectomy. Endoscopy.

[7] Bobkiewicz A, Krokowicz L, Banasiewicz T, Koscinski T, Borejsza-Wysocki M, Ledwosinski W (2014). Iatrogenic bile duct injury. A significant surgical problem. Assessment of treatment outcomes in the department’s own material. Pol Przegl Chir.

[8] Boerma D, Rauws EA, Keulemans YC, Bergman JJ, Obertop H, Huibregtse K (2001). Impaired quality of life 5 years after bile duct injury during laparoscopic cholecystectomy: a prospective analysis. Ann Surg.

[9] Landman MP, Feurer ID, Moore DE, Zaydfudim V, Pinson CW (2013). The long-term effect of bile duct injuries on health-related quality of life: a meta-analysis. HPB.

[10] Way LW, Stewart L, Gantert W, Liu K, Lee CM, Whang K (2003). Causes and prevention of laparoscopic bile duct injuries: analysis of 252 cases from a human factors and cognitive psychology perspective. Ann Surg.

[11] Nijssen MA, Schreinemakers JM, Meyer Z, van der Schelling GP, Crolla RM, Rijken AM (2015). Complications after laparoscopic cholecystectomy: a video evaluation study of whether the critical view of safety was reached. World J Surg.

[12] Buddingh KT, Nieuwenhuijs VB, van Buuren L, Hulscher JB, de Jong JS, van Dam GM (2011). Intraoperative assessment of biliary anatomy for prevention of bile duct injury: a review of current and future patient safety interventions. Surg Endosc.

[13] Dip FD, Asbun D, Rosales-Velderrain A, Lo Menzo E, Simpfendorfer CH, Szomstein S (2014). Cost analysis and effectiveness comparing the routine use of intraoperative fluorescent cholangiography with fluoroscopic cholangiogram in patients undergoing laparoscopic cholecystectomy. Surg Endosc.

[14] Ford JA, Soop M, Du J, Loveday BP, Rodgers M (2012). Systematic review of intraoperative cholangiography in cholecystectomy. Br J Surg.

[15] Schols RM, Connell NJ, Stassen LP (2015). Near-infrared fluorescence imaging for real-time intraoperative anatomical guidance in minimally invasive surgery: a systematic review of the literature. World J Surg.

[16] Verbeek FP, van der Vorst JR, Schaafsma BE, Hutteman M, Bonsing BA, van Leeuwen FW (2012). Image-guided hepatopancreatobiliary surgery using near-infrared fluorescent light. J Hepatobiliary Pancreat Sci.

[17] Mishra A, Behera RK, Behera PK, Mishra BK, Behera GB (2000). Cyanines during the 1990s: a review. Chem Rev.

[18] Rodrigues EB, Meyer CH, Mennel S, Farah ME (2007). Mechanisms of intravitreal toxicity of indocyanine green dye: implications for chromovitrectomy. Retina.

[19] Tagaya N, Shimoda M, Kato M, Nakagawa A, Abe A, Iwasaki Y (2010). Intraoperative exploration of biliary anatomy using fluorescence imaging of indocyanine green in experimental and clinical cholecystectomies. J Hepatobiliary Pancreat Sci.

[20] Cherrick GR, Stein SW, Leevy CM, Davidson CS (1960). Indocyanine green: observations on its physical properties, plasma decay, and hepatic extraction. J Clin Invest.

[21] Obana A, Miki T, Hayashi K, Takeda M, Kawamura A, Mutoh T (1994). Survey of complications of indocyanine green angiography in Japan. Am J Ophthalmol.

[22] Benya R, Quintana J, Brundage B (1989). Adverse reactions to indocyanine green: a case report and a review of the literature. Cathet Cardiovasc Diagn.

[23] Bjerregaard J, Pandia MP, Jaffe RA (2013). Occurrence of severe hypotension after indocyanine green injection during the intraoperative period. Case Rep.

[24] Schols RM, Lodewick TM, Bouvy ND, van Dam DA, Meijerink WJ, van Dam GM (2014). Near-infrared fluorescence laparoscopy of the cystic duct and artery in pigs: performance of a preclinical dye. J Laparoendosc Adv Surg Tech A.

[25] van den Bos J, Al-Taher M, Hsien SG, Bouvy ND, Stassen LP (2017). Near-infrared fluorescence laparoscopy of the cystic duct and cystic artery: first experience with two new preclinical dyes in a pig model. Surg Endosc.

[26] Srinivasan R, Singh M (2002). Development of biological tissue-equivalent phantoms for optical imaging. Indian J Exp Biol.

[27] Ballestriero R (2010). Anatomical models and wax venuses: art masterpieces or scientific craft works?. J Anat.

[28] Diana M, Soler L, Agnus V, D’Urso A, Vix M, Dallemagne B (2017). Prospective Evaluation of precision multimodal gallbladder surgery navigation: virtual reality, near-infrared fluorescence, and x-ray-based intraoperative cholangiography. Ann Surg.

[29] Graves C, Ely S, Idowu O, Newton C, Kim S (2017). Direct gallbladder indocyanine green injection fluorescence cholangiography during laparoscopic cholecystectomy. J Laparoendosc Adv Surg Tech A.

[30] Boogerd LSF, Handgraaf HJM, Huurman VAL, Lam HD, Mieog JSD, van der Made WJ (2017). The best approach for laparoscopic fluorescence cholangiography: overview of the literature and optimization of dose and dosing time. Surg Innov.

[31] Ankersmit M, van Dam DA, van Rijswijk AS, van den Heuvel B, Tuynman JB, Meijerink W (2017). Fluorescent imaging with indocyanine green during laparoscopic cholecystectomy in patients at increased risk of bile duct injury. Surg Innov.

[32] Gangemi A, Danilkowicz R, Elli FE, Bianco F, Masrur M, Giulianotti PC (2017). Could ICG-aided robotic cholecystectomy reduce the rate of open conversion reported with laparoscopic approach? A head to head comparison of the largest single institution studies. J Robot Surg.

[33] Zroback C, Chow G, Meneghetti A, Warnock G, Meloche M, Chiu CJ (2016). Fluorescent cholangiography in laparoscopic cholecystectomy: the initial Canadian experience. Am J Surg.

[34] Igami T, Nojiri M, Shinohara K, Ebata T, Yokoyama Y, Sugawara G (2016). Clinical value and pitfalls of fluorescent cholangiography during single-incision laparoscopic cholecystectomy. Surg Today.

[35] Dip F, Nguyen D, Montorfano L, Noste ME, Lo Menzo E, Simpfendorfer C (2016). Accuracy of near infrared-guided surgery in morbidly obese subjects undergoing laparoscopic cholecystectomy. Obes Surg.

[36] van Dam DA, Ankersmit M, van de Ven P, van Rijswijk AS, Tuynman JB, Meijerink WJ (2015). Comparing near-infrared imaging with indocyanine green to conventional imaging during laparoscopic cholecystectomy: a prospective crossover study. J Laparoendosc Adv Surg Tech A.

[37] Boni L, David G, Mangano A, Dionigi G, Rausei S, Spampatti S (2015). Clinical applications of indocyanine green (ICG) enhanced fluorescence in laparoscopic surgery. Surg Endosc.

[38] Dip F, Roy M, Lo Menzo E, Simpfendorfer C, Szomstein S, Rosenthal RJ (2015). Routine use of fluorescent incisionless cholangiography as a new imaging modality during laparoscopic cholecystectomy. Surg Endosc.

[39] Larsen SS, Schulze S, Bisgaard T (2014). Non-radiographic intraoperative fluorescent cholangiography is feasible. Dan Med J.

[40] Osayi SN, Wendling MR, Drosdeck JM, Chaudhry UI, Perry KA, Noria SF (2015). Near-infrared fluorescent cholangiography facilitates identification of biliary anatomy during laparoscopic cholecystectomy. Surg Endosc.

[41] Prevot F, Rebibo L, Cosse C, Browet F, Sabbagh C, Regimbeau JM (2014). Effectiveness of intraoperative cholangiography using indocyanine green (versus contrast fluid) for the correct assessment of extrahepatic bile ducts during day-case laparoscopic cholecystectomy. J Gastrointest Surg.

[42] Daskalaki D, Fernandes E, Wang X, Bianco FM, Elli EF, Ayloo S (2014). Indocyanine green (ICG) fluorescent cholangiography during robotic cholecystectomy: results of 184 consecutive cases in a single institution. Surg Innov.

[43] Schols RM, Bouvy ND, van Dam RM, Masclee AA, Dejong CH, Stassen LP (2013). Combined vascular and biliary fluorescence imaging in laparoscopic cholecystectomy. Surg Endosc.

[44] Buchs NC, Pugin F, Azagury DE, Jung M, Volonte F, Hagen ME (2013). Real-time near-infrared fluorescent cholangiography could shorten operative time during robotic single-site cholecystectomy. Surg Endosc.

[45] Spinoglio G, Priora F, Bianchi PP, Lucido FS, Licciardello A, Maglione V (2013). Real-time near-infrared (NIR) fluorescent cholangiography in single-site robotic cholecystectomy (SSRC): a single-institutional prospective study. Surg Endosc.

[46] Schols RM, Bouvy ND, Masclee AA, van Dam RM, Dejong CH, Stassen LP (2013). Fluorescence cholangiography during laparoscopic cholecystectomy: a feasibility study on early biliary tract delineation. Surg Endosc.

[47] Kaneko J, Ishizawa T, Masuda K, Kawaguchi Y, Aoki T, Sakamoto Y (2012). Indocyanine green reinjection technique for use in fluorescent angiography concomitant with cholangiography during laparoscopic cholecystectomy. Surg Laparosc Endosc Percutan Tech.

[48] Buchs NC, Hagen ME, Pugin F, Volonte F, Bucher P, Schiffer E (2012). Intra-operative fluorescent cholangiography using indocyanin green during robotic single site cholecystectomy. Int J Med Rob Comput Assist Surg.

[49] Ishizawa T, Kaneko J, Inoue Y, Takemura N, Seyama Y, Aoki T (2011). Application of fluorescent cholangiography to single-incision laparoscopic cholecystectomy. Surg Endosc.

[50] Ishizawa T, Bandai Y, Ijichi M, Kaneko J, Hasegawa K, Kokudo N (2010). Fluorescent cholangiography illuminating the biliary tree during laparoscopic cholecystectomy. Br J Surg.

[51] Aoki T, Murakami M, Yasuda D, Shimizu Y, Kusano T, Matsuda K (2010). Intraoperative fluorescent imaging using indocyanine green for liver mapping and cholangiography. J Hepatobiliary Pancreat Sci.

[52] Mitsuhashi N, Kimura F, Shimizu H, Imamaki M, Yoshidome H, Ohtsuka M (2008). Usefulness of intraoperative fluorescence imaging to evaluate local anatomy in hepatobiliary surgery. J Hepatobiliary Pancreat Surg.

[53] Liu YY, Liao CH, Diana M, Wang SY, Kong SH, Yeh CN (2017). Near-infrared cholecystocholangiography with direct intragallbladder indocyanine green injection: preliminary clinical results. Surg Endosc.

[54] Zarrinpar A, Dutson EP, Mobley C, Busuttil RW, Lewis CE, Tillou A (2016). Intraoperative laparoscopic near-infrared fluorescence cholangiography to facilitate anatomical identification: when to give indocyanine green and how much. Surg Innov.

[55] Verbeek FP, Schaafsma BE, Tummers QR, van der Vorst JR, van der Made WJ, Baeten CI (2014). Optimization of near-infrared fluorescence cholangiography for open and laparoscopic surgery. Surg Endosc.

[56] Kono Y, Ishizawa T, Tani K, Harada N, Kaneko J, Saiura A (2015). Techniques of fluorescence cholangiography during laparoscopic cholecystectomy for better delineation of the bile duct anatomy. Medicine.

[57] Ashitate Y, Stockdale A, Choi HS, Laurence RG, Frangioni JV (2012). Real-time simultaneous near-infrared fluorescence imaging of bile duct and arterial anatomy. J Surg Res.

[58] Landsman ML, Kwant G, Mook GA, Zijlstra WG (1976). Light-absorbing properties, stability, and spectral stabilization of indocyanine green. J Appl Physiol.

[59] Hilderbrand SA, Weissleder R (2010). Near-infrared fluorescence: application to in vivo molecular imaging. Curr Opin Chem Biol.

[60] Frangioni JV (2003). In vivo near-infrared fluorescence imaging. Curr Opin Chem Biol.

[61] Nguyen QT, Tsien RY (2013). Fluorescence-guided surgery with live molecular navigation–a new cutting edge. Nat Rev Cancer.

[62] Haritoglou C, Gandorfer A, Schaumberger M, Tadayoni R, Gandorfer A, Kampik A (2003). Light-absorbing properties and osmolarity of indocyanine-green depending on concentration and solvent medium. Invest Ophthalmol Vis Sci.

[63] Matsui A, Tanaka E, Choi HS, Winer JH, Kianzad V, Gioux S (2010). Real-time intra-operative near-infrared fluorescence identification of the extrahepatic bile ducts using clinically available contrast agents. Surgery.

[64] Peter C, Hongwan D, Kupfer A, Lauterburg BH (2000). Pharmacokinetics and organ distribution of intravenous and oral methylene blue. Eur J Clin Pharmacol.

[65] van den Bos J, Schols RM, Luyer MD, van Dam RM, Vahrmeijer AL, Meijerink WJ (2016). Near-infrared fluorescence cholangiography assisted laparoscopic cholecystectomy versus conventional laparoscopic cholecystectomy (FALCON trial): study protocol for a multicentre randomised controlled trial. BMJ Open.

[66] Figueiredo JL, Siegel C, Nahrendorf M, Weissleder R (2010). Intraoperative near-infrared fluorescent cholangiography (NIRFC) in mouse models of bile duct injury. World J Surg.

[67] Liu YY, Kong SH, Diana M, Legner A, Wu CC, Kameyama N (2015). Near-infrared cholecysto-cholangiography with indocyanine green may secure cholecystectomy in difficult clinical situations: proof of the concept in a porcine model. Surg Endosc.

[68] Zhu B, Sevick-Muraca EM (2015). A review of performance of near-infrared fluorescence imaging devices used in clinical studies. Br J Radiol.

[69] AV DS, Lin H, Henderson ER, Samkoe KS, Pogue BW (2016). Review of fluorescence guided surgery systems: identification of key performance capabilities beyond indocyanine green imaging. J Biomed Opt.

[70] Marshall MV, Rasmussen JC, Tan IC, Aldrich MB, Adams KE, Wang X (2010). Near-infrared fluorescence imaging in humans with indocyanine green: a review and update. Open Surg Oncol J.

[71] Orosco RK, Tsien RY, Nguyen QT (2013). Fluorescence imaging in surgery. IEEE Rev Biomed Eng.

