# Supplementary material for: Optimizing the image of fluorescence cholangiography using ICG: a systematic review and ex vivo experiments
Source: Surg Endosc. 2018 May 18;32(12):4820–32. doi: 10.1007/s00464-018-6233-x (PMC6208701; doi:10.1007/s00464-018-6233-x)
Supplement: Supplementary file 1 — Supplementary material 1 (DOCX 49 KB) [file 464_2018_6233_MOESM1_ESM.docx]

*Table 1 Characteristics of included studies*

| **Reference** | **No patients** | **Age**  (mean ± SD (range)) | **Male patients** (%) | **BMI** (mean ± SD (range)) | **ASA (%)** | **Type of**  **surgery*** | **Indication for surgery (%)** | **Used dye** | **Dose** (mg or adjusted to patient weight mg/kg) | **Timing** | **Used system** |
| --- | --- | --- | --- | --- | --- | --- | --- | --- | --- | --- | --- |
| Liu et al 2017 (53) | 46 | 49 ± 12.65 | 29 (63) | 27.16 ± 5.22 | Unknown | LC | Cholecystitis 25 (54)  Cholelithiasis 21 ((46) | ICG (DiagnoGreen®, Taiwan, ROC) | 1.25mg | During surgery | Karl Storz Endoscopes, Tuttlingen, Germany |
| Diana et al 2017 (28) | 54 | 51.6 ± 14.1 | 22 (37.9) | 28.6 ± 6.3 | Unknown | RC | Cholelithiasis 58 (100) | ICG (Infracyanine®, SERB, Paris, France) | 0.1 to 0.4 mg/kg | Upon anesthesia induction (45 to 60 minutes before incision) | Firefly® (Intuitive Surgical, Intuitive Inc., CA, USA) |
| Graves et al 2017 (29) | 11 | 16 | 4 (36.4) | Unknown | Unknown | LC | Acute cholecystitis 2 (18.2%)  Biliary colic 8 (72.7)  Biliary dyskinesia 1 (9.1) | ICG (IC-Green®, Akorn Inc., Lake Forest, IL) | 0.25mg | Once the fundus is grasped and retracted. (injection in gallbladder) | Stryker Corporation, Kalamazoo, MI |
| Boogerd et al 2017 (30) | 28 | 61 (20-76)  median (range) | 9 (32.1) | 5mg dose group 29 (22-37)  10 mg dose group  27 (22-36)  Median (range) | 5mg dose group ASA I 4 (25%)  ASA II 12 (75%)  10 mg dose group  ASA I 3 (25%)  ASA II 9 (75%) | LC | Cholelithiasis 25 (89.3)  Gallbladder polyp 2 (7.1)  Mirizzi syndrome 1 (3.6) | ICG (Pulsion Medical Systems, Munich, Germany) | 16 patients 5 mg  12 patients 10 mg | 30 minutes, 2, 4, or 6 hours prior to surgery | Karl Storz Endoscopes, Tuttlingen, Germany |
| Ankersmit et al 2017 (31) | 20 | 65 (26-82) | 13 (65) | 25.45 (16.8-38.0) | ASA I 7 (35%)  ASA II 10 (50%)  ASA III 3 (15%) | LC | Acute cholecystitis 7 (38)  Choledocholithiasis 7 (38)  Biliary pancreatitis 3 (17)  Cholangitis 1 (6) | ICG (Pulsion Medical Systems, Munich, Germany) | 0.2 mg/kg | Directly after the time-out procedure and induction of general anesthesia | Olympus, Tokyo, Japan |
| Gangemi et al 2016 (32) | 676 | 43.91 ± 16.12 | 180 (26.63) | 32.28 ±8.57 | ASA I 122 (18.24)  ASA II 378 (56.50)  ASA III 160 (23.92)  ASA IV 9 (1.35) | RC | Acute cholecystitis or gangrenous cholecystitis 131 (19.4)  Other 545 (80.6) | ICG | 2.5 mg | 45 minutes prior to operation | Firefly® (Intuitive Surgical, Intuitive Inc., CA, USA) |
| Zroback et al 2016 (33) | 12 | 53 (33-71) | 4 (33) | 28.9 ± 6 | 2 (1-3) mean (range) | LC | Chronic cholecystitis 3 (25) Biliary colic 6 (50) Previous cholecystostomy placement 2 (17) ERCP treated Choledocholithiasis 1 (8) | ICG | 3.75 mg | In the preoperative holding area | Unknown |
| Imagi 2016 (34) | 21 | 62 ± 11 (43–76)   <65 years 11 (52,4%)  >65 years 10 (47.6%) | 7 (33,3) | 23.9 ± 3.5 (18.8–32.4)   <25.0 kg/m^2^ 12 (57,1%)  >25.0 kg/m^2^ 9 (42,9%) | Unknown | SILS | Cholecystitis 2 (9.5)  Other 19 (90.5) | ICG (Diagnogreen; Diichi Sankyo Co., Tokyo, Japan) | 2.5 mg | After endotracheal intubation of the patient in the operating room. | Karl Storz Endoscopes, Tuttlingen, Germany |
| Dip et al 2016 (35) | 71 | Unknown | 29 (40,85) | Non-obese (BMI <30) 33 (46,5%) Obese (BMI>30) 38 (53,5%) | Unknown | LC | Acute cholecystitis 18 (25.4) Chronic cholecystitis 18 (25.4) Cholelithiasis 35 (49.3) | Indocyanine green (ICG) | 0.05 mg/kg | 1h preceding surgery | Karl Storz Endoscopes, Tuttlingen, Germany |
| Van Dam et al 2015 (36) | 30 | 49.7 ± 16.7 | 9 (30) | 27.5 ± 4.32 | ASA I 13 (43,33) ASA II 11 (36,67) ASA III 6 (20) | LC | Cholecystolithiasis 30 (100) | ICG (Pulsion Medical Systems, Munich, Germany) | 0.05 mg/kg | Immediately after induction of general anesthesia, and surgery began 10 ( ± 8.0) minutes after injection of ICG. | Olympus, Tokyo, Japan |
| Boni et al 2015 (37) | 52 | 53 ± 15 | 21 (40,4) | Unknown | Unknown | LC | Acute cholecystitis 35 (67.3) Cholelithiasis 17 (32.7) | ICG (Pulsion Medical Systems, Munich, Germany) | 0.04 mg/kg | At least 15 min before surgery | Karl Storz Endoscopes, Tuttlingen, Germany |
| Dip et al 2015 (38) | 45 | 49.24 ± 14.76 | 21 (46.7) | 28.35 ± 6.5 | Unknown | LC | Acute cholecystitis 17 (37.8) Chronic cholecystitis 5 (11.1) Cholelithiasis 22 (48.9) Gallbladder polyp 1 (2.2) | ICG (without iodine) | 0.05 mg/kg | One hour prior to surgery | Karl Storz Endoscopes, Tuttlingen, Germany |
| Larsen et al 2014 (39) | 35 | 48 (18-­74)  median (range) | 9 (25,7) | 28 (19­38) median (range) | Unknown | LC | Acute cholecystitis 7 (20)  Chronic cholecystitis 23 (65.7) Cholelithiasis 5 (14.3) | ICG | 2.5­-7.5 mg (0.05 mg/kg) | Directly after induction of anesthesia. | Olympus, Tokyo, Japan |
| Osayi et al 2015 (40) | 82 | 42.6 ± 13.7 | 18 (22) | 31.49 ± 8.2 | Unknown | LC | Chronic cholecystitis 18 (22) Cholelithiasis 49 (59.8)  Biliary pancreatitis 4 (4.9)  Biliary dyskinesia 9 (11) Choledocholithiasis 1 (1.2) Gallbladder polyp 1 (1.2) | Indocyanine green (IC-Green®, Akorn Inc., Lake Forest, IL, USA) ICG, (Pulsion Medical Inc., Irving, TX) | 2.5mg | Approximately 60 minutes prior to making a surgical incision | Stryker Endoscopy, San Jose, CA |
| Prevot et al 2014 (41) | 23 | 45 (18–81) | 2 (8.7) | Unknown | 2 (1–3) mean (range) | LC | Cholecystitis after antibiotic treatment 2 (9) Symptomatic cholecystolithiasis 14 (61)  ERCP treated choledocholithiasis 7 (30) | ICG  (Infracyanine®, SERB, Paris, France) | 0.5 mg/kg | After the induction of anesthesia | NIR 1® from Karl Storz Endoscopes, Tuttlingen, Germany |
| Dip et al 2014 (13) | 43 | 49.53 ± 14.35 | 21 (48.8) | 28.35 ± 8 | Unknown | LC | Acute cholecystitis 14 (32.6)  Chronic cholecystitis 5 (11.6) Cholelithiasis 22 (51.2)  Gangrenous cholecystitis 1 (2.3) | Indocyanine green (IC-Green®, Akorn Inc., Lake Forest, IL, USA) | 0.05mg/kg | 1h prior to surgery | D-light P-light-source unit (Karl Storz Endoscopes, Tuttlingen, Germany) |
| Daskalaki et al 2014 (42) | 184 | 42.4 (18-86) | 47 (25.5) | 32.1 (16.1-57.2) | I/II for 136 (74%)   III/IV for 48 (26%) | 122 RC 72 R-SILS | Acute or gangrenous cholecystitis 24 (13) Chronic inflammation and cholelithiasis 160 (87) | ICG | 2.5 mg | 45 minutes prior to the beginning of the surgical procedure. | Firefly® (Intuitive Surgical, Intuitive Inc., CA, USA) |
| Schols et al 2013 (43) | 30 | 53 (26–81) | 11 (36,7) | 26.7 (19.7–36.8) | Unknown | LC | Cholecystitis 8 (26.7)  Cholecystolithiasis 20 (66.7) Biliary pancreatitis 2 (6.7) | ICG  (Infracyanine®, SERB, Paris, France) | 2.5 mg | Directly after induction of anesthesia  15 patients: 2^nd^ dose of 2.5 mg of ICG when CVS for concomitant arterial and biliary fluorescence delineation | Karl Storz Endoscopes, Tuttlingen, Germany |
| Buchs et al 2013 (44) | 23 | 47.3 ± 11 | 11 (47.8) | 27±4.7 | 1.7 ± 0.6  mean ± SD | R-SILS | Cholelithiasis 23 (100) | Indocyanine green (ICG) | 2.5 mg | 30–45 min before incision | Firefly® (Intuitive Surgical, Intuitive Inc., CA, USA) |
| Spinoglio et al 2013 (45) | 45 | 48 (23–76)) | 12 (26) | 24.7 (19-43) | Unknown | R-SILS | Cholelithiasis/polyps 45 (100) | ICG | 2.5 mg | 30–45 min prior to the start of the operation | Firefly® (Intuitive Surgical, Intuitive Inc., CA, USA) |
| Schols 2013 (46) | 15 | 52 (27–76) median (range) | 4 (26.7) | 26.2 (19.7–31.6) median (range) | Unknown | LC | Cholecystitis 4 (26.7) Cholecystolithiasis 11 (73.3) | ICG  (Infracyanine®, SERB, Paris, France) | 2.5 mg | Directly after induction of anesthesia | Karl Storz Endoscopes, Tuttlingen, Germany |
| Kaneko et al 2012 (47) | 28 | 51 (22-78)  median (range) | 10 (35.7) | 20 (18 - 42)  median (range) | Unknown | 19 (67,9) LC 9 (32%) SILS | Cholelithiasis 27 (96.4) Gallbladder polyp 1 (3.6) | ICG (Diagnogreen; Daiichi Sankyo, Tokyo, Japan) | 0.05mg/kg; | 15 minutes before surgery under general anesthesia | Prototype fluorescence imaging system (Hamamatsu Photonics, Hamamatsu, Japan) |
| Buchs et al 2012 (48) | 12 | 47.1 (31–69)  median (range) | 4 (33.3) | 28 (20–39)  median (range) | Median ASA score was 2 (range 1–3) | R-SILS | Cholelithiasis 12 (100) | ICG | 2.5 mg | 30–45 minutes before the start of the case.   2^nd^ dose of 2.5 mg ICG if fluorescence was not detected in the liver 45 minutes after injection of the 1th dose or additional questions regarding perfusion | Firefly® (Intuitive Surgical, Intuitive Inc., CA, USA) |
| Ishizawa 2011 (49) | 7 | 45.3 (19-65) | 1 (14.3) | 20.4 (16.6–27.4)  mean (range) | Unknown | SILS | Unknown | ICG (Diagnogreen; Daiichi Sankyo, Tokyo, Japan) | 2.5 mg | after endotracheal intubation of the patient in the operating room | Prototype from Hamamatsu Photonics (Hamamatsu, Japan) |
| Ishizawa et al 2010 (50) | 52 | 59 (28 – 78)  median (range) | 29 (55.8) | 23.2 (18.1 – 32.9)  median (range) | Unknown | LC | Cholecystitis 2 (2.4)  Cholelithiasis 45 (86.5) Gallbladder polyp 5 (6.1) | ICG (Diagnogreen; Daiichi Sankyo, Tokyo, Japan) | 2.5 mg | 30min before the patient entered the operating room.  In a minority of patients ICG was administered after intubation in the operating room. | Prototype from Hamamatsu Photonics (Hamamatsu, Japan) |
| Aoki et al 2010 (51) | 14 | 61 (43–72) | 8 (57) | 4 (28.6%) patients 'obese' | Unknown | LC | Chronic cholecystitis with gallstones 14 (100) | ICG (Wako Pure Chemical Industries, Osaka, Japan) | 12.5 mg (5ml) | 30 min preoperatively | Prototype from Hamamatsu Photonics (Hamamatsu, Japan) |
| Tagaya et al 2010 (19) | 12 | Unknown | Unknown | Unknown | Unknown | 8 LC 4 OC | Unknown for laparoscopic patients  Acute cholecystitis 4 (100 % of open cholecystectomies) | ICG (Diagnogreen; Daiichi Sankyo, Tokyo, Japan) | 2.5 mg | 1–2 h before surgery  2^nd^ dose 2.5mg after identifying cystic artery | *‘a prototype infrared camera connected to a laparoscope’* |
| Mitsuhashi et al 2008 (52) | 5 | Unknown | Unknown | Unknown | Unknown | OC | Unknown  *'Patients 3 and 4 had mild inflammation of the gallbladder and Calot’s triangle'* | ICG | 2.5 mg | About 30 min before the surgery | infrared camera system (Photodynamic Eye; Hama- matsu Photonics, Shizuoka, Japan) |

*** Open cholecystectomy = OC, laparoscopic cholecystectomy = LC; Robot cholecystectomy = RC; single port = SILS; robot single port = R-SILS**
