# Supplementary material for: Optimizing the image of fluorescence cholangiography using ICG: a systematic review and ex vivo experiments
Source: Surg Endosc. 2018 May 18;32(12):4820–32. doi: 10.1007/s00464-018-6233-x (PMC6208701; doi:10.1007/s00464-018-6233-x)
Supplement: Supplementary file 2 — Supplementary material 2 (DOCX 136 KB) [file 464_2018_6233_MOESM2_ESM.docx]

*Table 2 Outcomes of included studies*

| **Reference** | **Visualisation cystic duct (%)** | **Visualisation common hepatic duct (%)** | **Visualisation common bile duct (%)** | **Visualisation junction cystic duct/common bile duct (%)** | **Visualisation cystic artery (%)** | **Total surgical time**  (in minutes, as mean ±SD unless otherwise indicated) |
| --- | --- | --- | --- | --- | --- | --- |
| Liu et al 2017 (53) | 84.7 | 73.9 | 78.2 | Unknown | Unknown | unknown |
| Diana et al 2017 (28) | Unknown | Unknown | Unknown | 53 (98,15) | Unknown | 132 ±39.75 |
| Graves et al 2017(29) | 10 (90.9) | 10 (90.9) | 10 (90.9) | 10 (90.9) | Unknown | Unknown |
| Boogerd et al 2017 (30) | 24 (96) | Unknown | Unknown | Unknown | Unknown | Unknown |
| Ankersmit et al 2017 (31) | 13 (72.2) | Unknown | 7 (38.9) | Unknown | Unknown | 82.5 (range 45-128) |
| Gangemi et al 2016 (32) | Unknown | Unknown | Unknown | Unknown | Unknown | Unknown |
| Zroback et al 2016 (33) | 12 (100) | 10 (83) | 6 (50) | Unknown | Unknown | 80 ± 31 |
| Imagi et al 2016 (34) | 10 (47.6) | Unknown | 17 (81.0) | 15 (71.4) | Unknown | 80 ± 29 (range 45–139) |
| Dip et al 2016 (35) | 71 (100) | 62 (87.3) | 50 (70.4) | Unknown | Unknown | Unknown |
| Van Dam et al 2015 (36) | 29 (96.7) | 26 (86,6) | Unknown | Unknown | Unknown | 71 ± 20.2 |
| Boni et al 2015 (37) | 52 (100) | 52 (100) | 52 (100) | 52 (100) | unknown | 54 ± 13 |
| Dip et al 2015 (38) | 44 (97.77) | 36 (80) | 27 (60 %) | Unknown | Unknown | 66.68 ± 19.17 |
| Larsen et al 2014 (39) | 35 (100) | 35 (100) | 35 (100) | 35 (100) | 29 (83%; 95% CI: 71­95%) | 43 (22-135)  median (range) |
| Osayi et al 2015 (40) | 78 (95.1) | Total 63 (76.8)  BMI <30 35 (81.4) BMI >30 28 (71.8) (p = 0.304) | Total: 57 (69,5)  BMI >30 33 (76.7)  BMI >30 24 (61.5)  (p = 0.135) | Total: 63 (76.8)   BMI <30 37 (86.1) BMI> 30 26 (66.7) (p = 0.038) | Unknown | 78.2 ± 30.7 |
| Prevot et al 2014 (41) | 23 (100) | Before dissection 3 (13)  After dissection 11 (48) | Before dissection 17 (74)  After dissection 18 (78) | Before dissection 11 (48)  After dissection 17 (74) | Unknown | 72 (40–200) mean (range) |
| Dip et al 2014 (13) | 42 (97.6) | 34 (43) | 25 (58.1) | Unknown | Unknown | 64.95 ± 17.43 |
| Daskalaki et al 2014 (42) | 180 (97.8) | Unknown | 173 (94) | 154 (83.6) | Unknown | 85.5 (30-230) 80  Mean (range) median |
| Schols et al 2013 (43) | 29 (97) | Unknown | 25 (83) | unknown | 13 (87) | 90 Median |
| Buchs et al 2013 (44) | Unknown | Unknown | Unknown | Unknown | Unknown | 85.2 ± 21.5 BMI < 25 70 ± 13.1  BMI >25 95 ± 20.4 |
| Spinoglio et al 2013 (45) | 42 (93) | 41 (91) | 40 (88) | 40 (88) | Unknown | 67 (35–110) mean (range) |
| Schols et al 2013 (46) | 15 (100) | Unknown | 15 (100) | Unknown | Unknown | Unknown |
| Kaneko et al 2012 (47) | 26 (93) | Unknown | 27 (96) | Unknown | 25 (89%) | 151 (98 - 343)  median (range) |
| Buchs et al 2012 (48) | 12 (100) | 10 (83.3) | 8 (66.7) | 7 (58.3) | Unknown | 85.7 (57– 125) Mean (range) |
| Ishizawa et al 2011 (49) | 5 (71.4) | Unknown | 7 (100) | 7 (100) | 4 (57.1) | Unknown |
| Ishizawa et al 2010 (50) | 52 (100) | Unknown | 52 (100) | 52 (100) | Unknown | 142 (91–366) median (range) |
| Aoki et al 2010 (51) | 10 (71.4) | Unknown | 10 (71.4) | Unknown | Unknown | Unknown |
| Tagaya et al 2010 (19) | 12 (100) | 12 (100) | 12 (100) | 12 (100) | 4 (33.3) | Unknown |
| Mitsuhashi et al 2008 (52) | 5 (100) | Unknown | Unknown | Unknown | Excellent 3 Good 1  Poor 1 | Unknown |
